# The corporate influence on food charity and aid: The “Hunger Industrial Complex” and the death of welfare

**DOI:** 10.3389/fpubh.2022.950955

**Published:** 2022-08-19

**Authors:** Martin Caraher, Sinéad Furey

**Affiliations:** ^1^Centre for Food Policy, City, University of London, London, United Kingdom; ^2^Department of Hospitality & Tourism Management, Ulster University, Coleraine, United Kingdom

**Keywords:** food charity, unhealthy food commodity industries, food policy, conflict of interest, greenwashing

## Abstract

There is an existing literature on how food companies, including the unhealthy food commodity industries, influence policy through a number of approaches. Direct approaches include lobbying and funding of research. Backdoor or indirect tactics used by food companies to demonstrate engagement include funding community groups, tactics previously used by the tobacco industry. Food industry support for food charities engaged in food donations is an area that has not received attention. This is another backdoor approach and one which may compromise more general public health policy. It is no surprise that the companies that engage in this can be largely fall under the rubric of unhealthy food commodity industries. This link is sometimes referred to as the “*hunger industrial complex”* and is based on the argument that an alliance exists between the food industry and the food banking movement. With rising levels of food insecurity there is pressure on the food system to donate food to charitable enterprises such as food banks and soups kitchens, which is often encouraged by government policy such as “*Good Samaritan legislation”*. Food businesses contribute surplus food and often promote it as part of their corporate social responsibility agenda. The argument presented here is not an anti-food charity one but one which challenges the development of charitable food aid as a system and a replacement for public policy. The reasons for this can be summarized under three headings: (1) such donation systems compromise the wider application of public food welfare and give the impression that food poverty is being addressed; (2) the links with food corporations provide a backdoor for influence on wider food policies; and (3) researchers taking money from food charities may be compromised by the direct and indirect relationships with companies. The focus in this paper is on the latter two issues; the first will be established as a context with work we have published elsewhere. This article draws on examples from the UK of how charities have linked with chocolate and soft drink companies. Examples include: “*For every Easter egg bought on the Cadbury Worldwide Hide, Cadbury will donate an Easter egg to a food bank in our network”;* a Coca-Cola initiative in December 2021 “*Win a meal, give a meal on-pack competition across Coca-Cola Original Taste and Coca-Cola Zero Sugar consumption packs, giving consumers the chance to win food-related vouchers, while donating to FareShar*e”; and an October 2021 initiative where “*McDonald's joins forces with FareShare to fund 1 million meals for UK families”*. These relationships go beyond companies donating surplus food to food charities such as food banks and pantries to encouraging consumers to buy their products with the promise that the company will contribute products to such charities or provide cash donations in return for the purchase of their product.

## Introduction

Many authors have identified the influence of the food industry on the formation of policy (1–3). This comes in a number of guises: directly funding research, funding nutrition research *via* industry-funded charities, lobbying through formal means, use of their financial power as major contributors to employment and food supply, and more informal approaches contributing to helping alleviate social problems such as hunger (4–8). These latter approaches are sometimes referred to as backdoor approaches and are often used by the unhealthy food commodity industries (UFCIs), tactics previously used by the tobacco industry. The intent is to build community support and identity with a product or company through fundings of community groups. Such backdoor tactics can be used by food companies to demonstrate engagement. An example from the USA is soft drink manufacturers' funding of the “National Association for the Advancement of Colored People” (NAACP), resulting in local branches of the organization opposing the imposition of soda taxes (7). Akin to academics taking money direct from the food industry, the acceptance of industry monies by community groups appears to help develop a positive attitude to the industry and the associated products (7, 9). The focus of this article is on the UFCIs as case studies, we recognize there is a broader debate to be had with respect to the wider food industry and links and food donations, some of which are less clear and more problematic and introduce gray areas. This can include supermarkets who donate fresh fruit and vegetables to food charities. This article focuses on the case of corporate philanthropy/food charity as an example of a problematic actor linking with an ostensibly beneficial actor and the possible conflicts of interest that can arise from such relationships (7, 9). This is an area that has not received much attention. Such developments can be seen as backdoor approaches and one which may ultimately compromise more general public health policy. The ultimate outcome of such an approach is referred to as the corporate capture of public health where industry influence dominates the policy agenda (10).

In many instances of food policy, the influence is less visible and benign—but potentially no less toxic for all of that. The association with food charities lends a “*halo effect”* to companies seeking to present a positive image. However, it is important to note that “*greenwashing”* occurs if there is a lack of coherence between what a firm communicates and its actions (10). Greenwashing is likely to eradicate consumer trust, which is a key requisite for customers when engaging with food companies. According to Power, the main food aid charities in the UK by accepting funding from companies such as Tesco, ASDA and British Gas have strengthened their alliances with “*corporate Britain”* (11) (p. 141). Andy Fisher in his book “Big Hunger” and in his blogs calls this link the “*hunger industrial complex”* and argues that an alliance exists between the food industry and the food banking movement (12, 13). With rising levels of food insecurity there is pressure on the food system to donate food to charitable enterprises such as food banks and soup kitchens. Such developments are often encouraged by government policy including “*Good Samaritan legislation”* (14). This essentially exempts them from any subsequent harm caused by the food such as food poisoning and food allergens. Additionally by providing space for charity to deliver food to those in need, welfare provision can be scaled back, saving monies in the public purse (11, 14, 15).

Food businesses contribute food and often promote it as part of their corporate social responsibility agenda. This is generally food that is surplus to requirements due to oversupply, food close to being out of date and /or food that is not readily saleable by reason of appearance, freshness, grade, size, or cosmetic blemishes. This can also include food in the hospitality sector that is left unsold at the end of the day's trading due to inefficient/irresponsible food ordering. This allows companies to claim a “*halo* effect” as they can claim “*good deeds”* while avoiding spending money on recycling or sending food to land fill. There is an abundant literature on the (in)appropriateness of the use of surplus or waste food to feed people who are food insecure and arguments related to how this contributes to the roll back of the state and the citizens' right to food. The former UN Special Rapporteur on food poverty said “*Foodbanks are a testimony to the failure of public authorities to deliver on the right to food and should be neither a permanent feature nor a substitute for more robust social programs. Food assistance in the form of the right to social security, such as cash transfers, food stamps or vouchers, can be defined in terms of rights, whereas foodbanks are charity-based and depend on donations and good will*” (16). As such, we need to realize the aspiration that charitable food aid should not be normalized as a response to food insecurity and that a “cash first” response better serves citizens' and policymakers' dignity aspirations (16).

The focus in this perspective article is on the relationship being developed by the food industry and especially UFCIs with the charity sector and the increasing use of direct forms of influence such as customer donations and/or direct provision of food or meals. This can contribute to conflicts of interest at two levels: the first is at the individual level for researchers who conduct research for food charities with strong links with UFCIs; and secondly at the level of general policy making for the center or department within which the researcher works. As already referred to above, Andy Fisher (12) in his book (chapter 4, p. 105–142) details how links between a university department and food welfare work with food charities later led to wider conflicts in wider public health nutrition policy. He broadens out the discussion to food charities and their links with the UFCIs. These conflicts arose over the issue of setting limits on the amount of sugar-based drinks people could access or buy using welfare benefits. Within food choice and poverty policy, the issue of choice assumes importance as opposed to tacking structural influences (17). In many ways those choices are seen as being limited and dictated by the dominant food system, so why blame “the poor” (18)? Fisher details how the major advocacy agency for food poverty opposed the setting of limits for unhealthy food (such as soft drinks) under food welfare or nutrition supplementation schemes such as the US Supplemental Nutrition Assistance Program, commonly known as SNAP (11). But, when it comes to issues of public health nutrition policy and obesity setting limits on unhealthy food products is seen as key; hence the conflict.

Recent examples of links with UFCIs are taken from the two main charity providers of food in the UK: FareShare (FS) and the Trussell Trust (TT). FareShare is a food redistribution charity that operates throughout the UK with the overarching aim to improve access to good quality, healthy food for low-income, vulnerable individuals while supporting the food industry to reduce the environmental impact of food waste. It acts as a “*wholesaler”* to groups providing food to those in need such as community kitchens, breakfast clubs etc. The Trussell Trust, a Christian charity, consists of a network of 1,500 franchised food banks. It started by relying on donations from the public, but more and more is developing links with food retailers at the local level. So, for example, they now work with the UK major supermarket, Tesco so that food waste/surplus from its stores will be donated to local food charities.

## The cases

We chose UFCIs as case studies and while these may represent the extreme of the links with unhealthy food commodities, we justify this on the basis that this is a perspective article and recognize the need for further studies on the links with more general retailers who may donate a mix of healthy and unhealthy products. The argument is still that located in the research which shows that such initiatives do little to tackle household food insecurity that the major beneficiaries are large corporations and companies (3). Details of websites when accessed and screen shots can be found in the Supplementary Data. The first example is a link between Cadbury (owned by Mondeléz International) and TT of a donation in kind for every piece of chocolate bought (19). On the TT website (20) the headline is “*Join our fight to end hunger and poverty this Easter: We're joining forces with Cadbury once again to make Easter special for all”*. And further on “*For every Easter egg bought on the Cadbury Worldwide Hide, Cadbury will donate an Easter egg to a food bank in our network”* (19). The link is with Easter and therefore symbolic of giving and celebration after the Lenten period of abstinence. The claim to end poverty is a major headline. There is a similar promotion for Christmas with a secret Santa approach where the promise on the Cadbury's website is “*When you send a Cadbury Secret Santa to someone you love this Christmas, Cadbury will donate another chocolate bar on your behalf to a food bank in the Trussell Trust network*” (20). Again, the connection is with a time of giving and notions of people going without at a time of celebration.

Two further examples of these corporate links between FS and Coca Cola and McDonald's follow. A joint Coca-Cola/FareShare initiative in December 2021 was for the “Real Magic at Christmas campaign” (21). The headline message on the website was “*Win a meal, give a meal on-pack competition across Coca-Cola Original Taste and Coca-Cola Zero Sugar consumption packs, giving consumers the chance to win food related vouchers, while donating to FareShar*e”. The next example is a link between McDonald's and FareShare. In October 2021 “*McDonald's joins forces with FareShare to fund 1 million meals for UK families”* (22). The McDonald's website outlining the initiative contains endorsements from the CEO of FareShare and from UK celebrity footballer and FareShare ambassador Marcus Rashford. The latter said “*Since joining the Child Food Poverty Taskforce, McDonald's has continued to show great commitment to the cause, and this Christmas period is no different. A time that should be met with joy and togetherness, will be met with stress, anxiety, and sadness for many struggling families. I'm so pleased and thankful to McDonald's for taking a proactive step in alleviating some of that worry, by partnering with FareShare on the distribution of 5 million vital meals”*. The annual report of FS for the year ended 31st March 2021 identifies McDonald's as a new corporate partnership, along existing donors including Tesco, ASDA, the Co-Op, Waitrose/John Lewis partnership, Sainsbury's (all food retailers) and the Compass Group (catering), Pepsi.Co as well as contributions from government during the COVID lockdown (23).

There are many links to food companies on the TT website including Unilever, Mars Food, Asda, Tesco, Waitrose and these are declared in their Annual Report to the Charity Commission (24). The final example comes from new links that the TT has developed with Deliveroo, the online delivery company (25). The UK-wide partnership enables customers to add a round-up donation to their in-app food orders, with all proceeds going to the Trussell Trust's food banks. Deliveroo employees can also volunteer for the charity, as a result of the partnership. This partnership is with a company which has been constantly criticized by unions and workers for the conditions of employment and underpayment, with many who work in the sector suffering from food poverty (26, 27). Those who work in the food production/retail industry should, one would assume, be protected from food insecurity, yet our food sector workers have experienced higher levels of food insecurity despite being employed.

## Discussion

We have written elsewhere (14) about the limits and potential limits of using surplus food of food hungry and insecure people, in summary:

- Analysis of the benefits and drawbacks of the use of surplus food to feed food insecure people highlights how this practice undermines calls for direct actions to both reduce the production of surplus food by considering the wider issue of overproduction in the food industry as well as the wastage in food distribution chains, and to address upstream drivers of food insecurity and ensure the right to food.
- Recommendations call for civil society and policymakers to focus on systemic solutions to both food waste and household food insecurity as separate entities.
- While the redistribution of food waste to emergency food aid providers offers immediate relief in the short-term, there is no evidence to show that it addresses food insecurity.
- There is evidence from other countries that the use of food waste for emergency food aid “*depoliticizes”* hunger and allows governments not to address the gap between income and food.

As noted earlier the work by Azadian et al. points out that the benefits of company links with food charities are not food insecure households but large companies and corporations (3). Oxfam similarly reports that the major beneficiaries in the COVID lockdown were “*companies in the food, pharma, energy, and tech sectors”* that benefited most (28). These debates are, of course not new, with a history of company involvement from tobacco to pharmaceuticals (29). Navarro in his examination of public policy responses to the COVID-19 pandemic by governments was weakened by cuts to public health services and that the large trans-national companies were the major beneficiaries. He claims that such moves are the result of “neoliberal policies, such as the politics of austerity” (30). There is also the wider danger of association with companies who promote low wages and lobby against healthy food regulation (3, 31).

At the risk of being called killjoys and being accused of denying those in poverty access to soft drinks or chocolate, we have argued elsewhere for more comprehensive welfare policies to tackle food insecurity (13, 14). The focus here is in the potential wider fallouts from links with UFCIs and how these might compromise wider public health programmes. In the case study examples both FareShare and McDonald's refer to their membership of the *Child Food Poverty Taskforce*. This is an initiative set up by the FareShare ambassador and celebrity footballer, Marcus Rashford, who has called for wider and long-term approaches by government to food poverty. He was instrumental during the COVID lockdowns in ensuring that schoolchildren were fed while schools were shut and in extending the right to free school meals to all children in need (32). It is estimated that his intervention on behalf of FareShare resulted in an estimated £20 m of additional donations to the food poverty charity (33). This is clearly great work but the extent to which companies such as McDonald's and Coca-Cola are basking in the halo effect is unclear. Some might argue that they are just exercising their function as good corporate citizens, others that there is a danger by association of compromising public health ideals.

The case examples described above and the links with UFCIs run the risk of wider conflict when it comes to discussions over the formation of public health nutrition policy. The cases described above have gone beyond companies donating surplus food to food charities such as food banks and pantries to encouraging consumers to buy their products with the promise that the company will contribute products to such charities or provide cash donations in return for the purchase of their product. The case examples show a move beyond donations of surplus food to direct contributions by companies to food charities often based on a percentage of customer purchases or a direct contribution (often a top-up or rounding up of the price, say from £2.75 to £3.00 resulting in a 25 pence donation) *via* the company which is then directed to the food charity. These are generally found to be reported under companies' corporate social responsibility headings (34).

There are arguments that there is nothing such as “*clean money”* and that all funding is tainted; the best that we can do is aim for transparency. Hennessey et al. call for complete transparency in nutrition research and Pizzo et al. add that there is a need to balance the conflicts between individual and institutional conflicts of interest (35, 36). Although both are referring to direct funding by industry interests we argue there is need to extend this to indirect influence and contact with industry players through intermediary groups such as food charities. In many ways the issue is a sin of omission as opposed to a sin of commission (12). Declarations of interests (DOIs) need to be broader in their scope to capture such relationships. The problem is not just one for these individual researchers who might be funded by or have access to food charity records working on food poverty or the nutrition content of food parcels but can become an issue for colleagues in the same department working on issues related to wider nutrition policies as shown by Fisher (11). What happens when wider policy discussions take place over issues such as sugar and fat taxes and subsidies? How do researchers or departments who have worked for charities with links to the UFCI declare this, or are they even aware of it? We are not suggesting that the links are direct in that companies such as McDonald's or Cadbury's (Mondeléz) fund research activities but by association and they may be involved in wider greenwashing as was shown by Fisher (12). This is important for departments of public health and nutrition (37) who should be mindful to scrutinize any collaborations between, for example, industry and charitable organizations and/or academia to eradicate the potential for greenwashing of CSR reporting. Of course it is important to recognize that it is not just the unhealthy food commodity industries that engage in such paractices but also sectors such as the food retail industry. Möller calls this the “*marketisation of food charity”* (38) (p. 26). This needs further examination as many of the foods donated here may be equally unhealthy often ultra processed and high in sugar, fat and salt (15, 35).

In terms of COIs, journals should extend the question on links with the food industry to include a declaration of indirect links such as a charity working with UFCIs. We are not suggesting that researchers should not work for charities who accept industry money but that there be more transparency around such work and partnerships. This moves beyond the recommendations put forward by Gottlieb et al. in how journals should handle conflicts of interest to identifying links with industry players through third party actors such as charities even if no direct financial contribution to the research is evident (39).

## Data availability statement

The original contributions presented in the study are included in the article/Supplementary material, further inquiries can be directed to the corresponding author/s.

## Author contributions

This work picks up on and develops work we have previously carried out on the role of commercial food aid to charities. MC and SF worked on the initial idea and identified case reports which would fit in a perspective article and collaborated on the refinement of the analysis and the narrative. Initial analysis was carried out by MC and checked by SF. Both authors contributed to the article and approved the submitted version.

## Conflict of interest

The authors declare that the research was conducted in the absence of any commercial or financial relationships that could be construed as a potential conflict of interest.

## Publisher's note

All claims expressed in this article are solely those of the authors and do not necessarily represent those of their affiliated organizations, or those of the publisher, the editors and the reviewers. Any product that may be evaluated in this article, or claim that may be made by its manufacturer, is not guaranteed or endorsed by the publisher.
