# Supplementary material for: The corporate influence on food charity and aid: The “Hunger Industrial Complex” and the death of welfare
Source: Front Public Health. 2022 Aug 19;10:950955. doi: 10.3389/fpubh.2022.950955 (PMC9437921; doi:10.3389/fpubh.2022.950955)
Supplement: Supplementary file 1 [file Data_Sheet_1.docx]

Supplementary Material

**Copies (Screenshots) of web pages**

A link between Cadbury (owned by Mondeléz) and TT of a donation in kind for every piece of chocolate bought. On the TT website ([**https://www.trusselltrust.org/cadbury/**](https://www.trusselltrust.org/cadbury/) ) the headline is ‘*Join our fight to end hunger and poverty this Easter, We’re joining forces with Cadbury once again to make Easter special for all’*.

Accessed 20/4/2022

Cadbury’s website ([**https://www.cadburygiftsdirect.co.uk/secret-santa-trussell-trust**](https://www.cadburygiftsdirect.co.uk/secret-santa-trussell-trust) ) ‘*When you send a Cadbury Secret Santa to someone you love this Christmas, Cadbury will donate another chocolate bar on your behalf to a food bank in the Trussell Trust network*’.

Accessed 20/4/2022

A joint Coca-Cola/FareShare initiative in December 2021 was FS partnership with Coca-Cola for the ‘Real Magic at Christmas campaign’ ([**https://fareshare.org.uk/news-media/news/fareshare-partners-with-coca-cola-for-real-magic-at-christmas-campaign/**](https://fareshare.org.uk/news-media/news/fareshare-partners-with-coca-cola-for-real-magic-at-christmas-campaign/))

Accessed 20/4/2022

A link between McDonalds and FareShare. In October 2021 ‘*McDonald’s joins forces with FareShare to fund 1 million meals for UK families’*

https://fareshare.org.uk/news-media/news/mcdonalds-joins-forces-with-fareshare-to-fund-1-million-meals-for-uk-families/

Accessed 20/4/2022

TT has developed with Deliveroo the online delivery company (<https://www.trusselltrust.org/get-involved/partner-with-us/strategic-partners/deliveroo/>

Accessed 20/4/2022
